# Reply: ‘Behr syndrome’ with *OPA1* compound heterozygote mutations

**DOI:** 10.1093/brain/awu235

**Published:** 2014-08-21

**Authors:** Patrick Yu-Wai-Man, Patrick F. Chinnery

**Affiliations:** 1 Departments of Neurology and Ophthalmology, Royal Victoria Infirmary, Newcastle upon Tyne, UK; 2 Wellcome Trust Centre for Mitochondrial Research, Institute of Genetic Medicine, Newcastle University, Newcastle upon Tyne, UK

Sir,

The current report by Carelli and colleagues is a timely contribution the literature on autosomal dominant optic atrophy (DOA) (Carelli *et al.*, 2014). Similar to a recently published Letter to the Editor in *Brain* by Bonneau *et al.* (2014), they describe the intriguing association of a Behr-like phenotype in an Italian family harbouring presumed compound heterozygous *OPA1* mutations. The majority of patients with DOA harbour single heterozygous mutations within the *OPA1* gene and haploinsufficiency is widely regarded as the major disease mechanism that precipitates retinal ganglion cell loss and progressive visual failure (Lenaers *et al.*, 2012). However, several fundamental questions remain unanswered, in particular, the wide intra- and inter-familial variability in disease severity that is observed not only in relation to the degree of visual loss, but also with the increasing realisation that a sizeable proportion of *OPA1* mutation carriers will develop significant neurological complications during their lifetime (Yu-Wai-Man *et al.*, 2010). We must now reconcile these diverging genotypes and phenotypes in a disorder that has until recently been viewed as being limited to the optic nerve with a straightforward autosomal dominant mode of inheritance (Yu-Wai-Man and Chinnery, 2014).

Seven unrelated families presenting with the classical manifestations of Behr syndrome have now been reported and the development of this syndromic form of DOA has been ascribed to the deleterious synergistic consequences of compound heterozygous *OPA1* mutations (Schaaf *et al.*, 2011; Bonifert *et al.*, 2014; Bonneau *et al.*, 2014; Carelli *et al.*, 2014). In six of these cases, a truncative *OPA1* mutation occurred in *trans* with the c.1146A>G (p.Ile382Met) missense variant, which involves the protein’s critical catalytic GTPase domain. *What important insight have we gained so far from these atypical DOA families with Behr-like phenotypes?* The truncative *OPA1* mutation is presumably resulting in haploinsufficiency and the working hypothesis being put forward is that the amino acid substitution (p.Ile382Met) induced by the c.1146A>G variant is somehow exerting a modulatory influence on the multiple cellular functions mediated by the protein’s GTPase domain (Schaaf *et al.*, 2011; Bonifert *et al.*, 2014; Bonneau *et al.*, 2014; Carelli *et al.*, 2014). The c.1146A>G variant is relatively rare, being present at a frequency of <0.1% in large public genomic databases (Bonifert *et al.*, 2014). Interestingly, the majority of familial carriers are clinically asymptomatic with no subclinical evidence of optic nerve dysfunction when harbouring this specific *OPA1* variant in isolation (even in the compound homozygous state). The overall conclusion therefore is that the c.1146A>G variant is relatively benign in the absence of another truncative *OPA1* mutation, at least in the families with Behr-like phenotypes that have been reported so far. In two other reports, the c.1146A>G variant was linked to a mild form of optic neuropathy in a French and a German proband (Schimpf *et al.*, 2008; Ferre *et al.*, 2009). Carelli and colleagues are also investigating an Italian patient with optic atrophy and ataxia who seems to be heterozygous for the c.1146A>G variant on its own (Carelli *et al.*, 2014). The obvious question is whether the c.1146A>G variant is causing the disease phenotype *per se*; whether there is another pathogenic *OPA1* variant that has been missed including deep intronic mutations; or whether this c.1146A>G variant is a red herring with another nuclear gene besides *OPA1* being the real cause.

The functional consequences of the c.1146A>G variant clearly needs to be investigated further to clarify its contributory role in the development and progression of clinical features in patients with DOA. Although these findings should be viewed as preliminary, fibroblasts carrying the c.1146A>G variant had reduced oxidative phosphorylation efficiency and about a 25% reduction in complex IV activity. The mitochondrial network was also dysmorphic with the presence of swollen mitochondrial segments pointing towards an underlying fusion-fission imbalance (Chevrollier *et al.*, 2008). Although the c.1146A>G variant could be an important clue in understanding why some patients are more severely neurologically affected, the mechanistic impact of bi-allelic *OPA1* mutations in the emergence of syndromic DOA+ phenotypes needs further consideration. In one of the four unrelated affected children reported by Bonneau and colleagues, the second putative pathogenic *OPA1* allele was the c.1204G>A (p.Val402Met) variant, and not the c.1146A>G (p.Ile382Met) variant (Bonneau *et al.*, 2014). In our original multicentre study of 104 patients with DOA+ (Yu-Wai-Man *et al.*, 2010), the c.1146A>G variant was also not detected, clearly implicating other intralocus *OPA1* disease-modifiers or alternative mechanisms (besides bi-allelic mutations) for the development of multisystemic neurological involvement. The *OPA1* story is turning out to be a fascinating one, with new tantalizing twists at every turn, and each time providing us with a little piece of the biological puzzle that accounts for a wide range of seemingly disparate human pathologies.

## Funding

P.Y.W.M. is a Medical Research Council (MRC, UK) Clinician Scientist. P.Y.W.M. also receives funding from Fight for Sight (UK) and the UK National Institute of Health Research (NIHR) as part of the Rare Diseases Translational Research Collaboration. P.F.C. is a Wellcome Trust Senior Fellow in Clinical Science and a UK National Institute of Health Research (NIHR) Senior Investigator who also receives funding from the MRC (UK) and the UK NIHR Biomedical Research Centre for Ageing and Age-related disease award to the Newcastle upon Tyne Hospitals NHS Foundation Trust.
